# 2,2′-Diamino-5,5′-dimethyl-4,4′-bi-1,3-thia­zolium tetra­chlorido­zincate(II)

**DOI:** 10.1107/S1600536809042007

**Published:** 2009-10-28

**Authors:** Akram Hosseinian, Ali Reza Mahjoub

**Affiliations:** a Engineering Science, University of Tehran, Tehran, 11365-4563, Iran; b Department of Chemistry, Tarbiat Modares University, Tehran, 14115-175, Iran

## Abstract

In the dianion of the title compound, (C_8_H_12_N_4_S_2_)[ZnCl_4_], the Zn^II^ ion is in a slightly distorted tetra­hedral environment. In the cation, the mean planes of the thia­zole rings form a dihedral angle of 67.81 (6) Å. In the crystal structure, anions and cations are linked into a three-dimensional network *via* inter­molecular N—H⋯Cl hydrogen bonds.

## Related literature

For the potential applications of metal-organic coordination compounds as anti­tumor drugs, polymers and luminescent materials, see: Hosseinian & Mahjoub (2006 ▶). For bond-length data, see: Allen *et al.* (1987 ▶). 
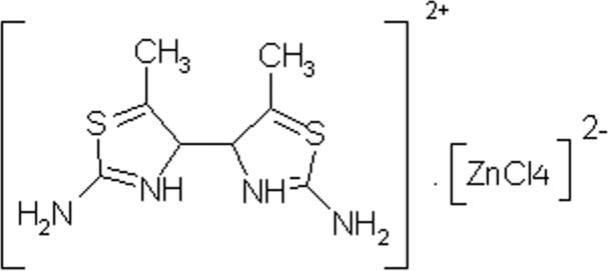


## Experimental

### 

#### Crystal data

(C_8_H_12_N_4_S_2_)[ZnCl_4_]
*M* *_r_* = 435.51
Triclinic, 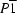

*a* = 8.9149 (6) Å
*b* = 9.6487 (7) Å
*c* = 11.7361 (8) Å
α = 65.754 (5)°
β = 89.126 (5)°
γ = 62.496 (5)°
*V* = 797.22 (12) Å^3^
*Z* = 2
Mo *K*α radiation
μ = 2.46 mm^−1^
*T* = 120 K
0.30 × 0.30 × 0.25 mm

#### Data collection

Bruker SMART 1000 CCD area-detector diffractometer
Absorption correction: multi-scan (*SADABS*; Sheldrick, 1996 ▶) *T*_min_ = 0.484, *T*_max_ = 0.535
6168 measured reflections
3368 independent reflections
3152 reflections with *I* > 2σ(*I*)
*R*_int_ = 0.022

#### Refinement

*R*[*F* ^2^ > 2σ(*F* ^2^)] = 0.029
*wR*(*F* ^2^) = 0.077
*S* = 1.05
3368 reflections
173 parameters
H-atom parameters constrained
Δρ_max_ = 0.66 e Å^−3^
Δρ_min_ = −0.82 e Å^−3^

### 

Data collection: *SMART* (Bruker, 1998 ▶); cell refinement: *SAINT-Plus* (Bruker, 1998 ▶); data reduction: *SAINT-Plus*; program(s) used to solve structure: *SHELXTL* (Sheldrick, 2008 ▶); program(s) used to refine structure: *SHELXTL*; molecular graphics: *SHELXTL*; software used to prepare material for publication: *SHELXTL*.

## Supplementary Material

Crystal structure: contains datablocks I, global. DOI: 10.1107/S1600536809042007/lh2898sup1.cif

Structure factors: contains datablocks I. DOI: 10.1107/S1600536809042007/lh2898Isup2.hkl

Additional supplementary materials:  crystallographic information; 3D view; checkCIF report

## Figures and Tables

**Table:** 

Zn1—Cl1	2.2531 (6)
Zn1—Cl3	2.2642 (6)
Zn1—Cl2	2.2707 (6)
Zn1—Cl4	2.2788 (6)

**Table:** 

Cl1—Zn1—Cl3	110.69 (3)
Cl1—Zn1—Cl2	110.34 (2)
Cl3—Zn1—Cl2	106.06 (2)
Cl1—Zn1—Cl4	111.25 (3)
Cl3—Zn1—Cl4	108.19 (3)
Cl2—Zn1—Cl4	110.16 (3)

**Table 2:** Hydrogen-bond geometry (Å, °)

*D*—H⋯*A*	*D*—H	H⋯*A*	*D*⋯*A*	*D*—H⋯*A*
N1—H1*A*⋯Cl2^i^	0.87	2.78	3.440 (3)	133
N2—H2*A*⋯Cl4^ii^	0.89	2.79	3.487 (2)	137
N3—H3*A*⋯Cl4^iii^	0.87	2.50	3.322 (3)	156
N3—H3*B*⋯Cl2^iv^	0.83	2.36	3.196 (3)	179
N4—H4*A*⋯Cl1^v^	0.87	2.44	3.280 (3)	162
N4—H4*B*⋯Cl3^ii^	0.89	2.38	3.189 (2)	151

Symmetry codes: (i) 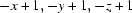; (ii) 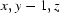; (iii) 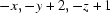; (iv) 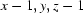; (v) 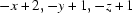.

**Fig. 1.**
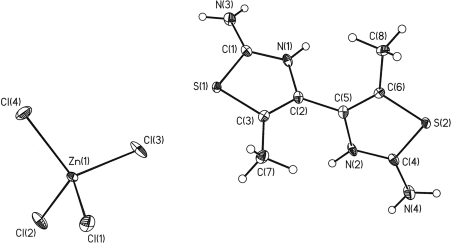
The molecular structure of (I). Displacement ellipsoids are drawn at the 30% probability level and H atoms are shown as small spheres of arbitrary radii.

**Fig. 2.**
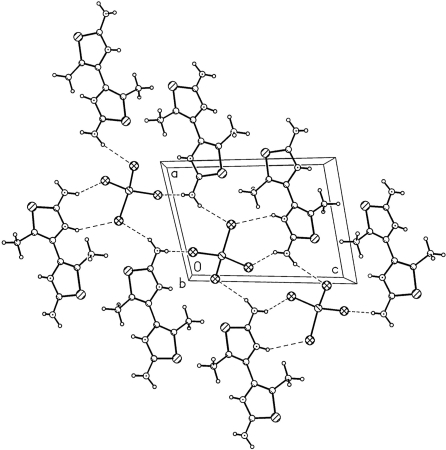
Part of the crystal structure with dashed lines indicating hydrogen bonds. Only H atoms involved in hydrogen bonds are shown.

## supplementary crystallographic information

### Comment

In coordination chemistry there are many studies on the interaction of Zn^II^ ions with biomolecules (Hosseinian & Mahjoub, 2006: and references cited therein). Coordination between an organic ligand and Zn^II^ ions improves or modifies the properties of biological molecules. In the human body the second abundant trace metal is zinc and it can be considered as a non toxic metal. The presence of zinc is vital to 300 enzyme structures, regulations and catalytic actions. As part of our research in the field of Zn^II^ complexes of organic molecules the crystal structure of the title complexes is presented herein.

The asymmetric unit of the title compound is shown in Fig. 1. The bond lengths have normal values (Allen *et al.*,  1987). In the crystal structure, anions and cations are linked into a three-dimensional network via intermolecular N-H···Cl hydrogen bonds. In addition, there are fairly close intermolecular S···Cl contacts ca. 3.24Å.

### Experimental

To a methanol solution of ZnCl_2_ (1 mmol, 0. 136 g) was added, 2,2'-Diamino-5,5'-Dimethyl-4,4'-bithiazole (dadmbtz) (1 mmol, 0. 226 g). The mixture was refluxed for 2 h. The solution was cooled and filtrate was slow evaporated at room temperature. After 12 days, yellow block shaped crystals of the title compound were obtained.

### Refinement

The hydrogen atoms boned to N atoms were located in difference Fourier maps and refined in 'as found' positions in a riding-model approximation with *U*_iso_(H) = 1.2U_eq_(N). H atoms boned to C atoms were placed in calculated positions and refined in a riding-model approximation with *U*_iso_(H) = 1.2U_eq_(C) or 1.5U_eq_(C) for methyl H atoms.

### Crystal data

**Table:**

(C_8_H_12_N_4_S_2_)[ZnCl_4_]	*Z* = 2
*M**_r_* = 435.51	*F*(000) = 436
Triclinic, *P*1	*D*_x_ = 1.814 Mg m^−^^3^
Hall symbol: -P 1	Mo *K*α radiation, λ = 0.71073 Å
*a* = 8.9149 (6) Å	Cell parameters from 5086 reflections
*b* = 9.6487 (7) Å	θ = 2.5–28.0°
*c* = 11.7361 (8) Å	µ = 2.46 mm^−^^1^
α = 65.754 (5)°	*T* = 120 K
β = 89.126 (5)°	Prism, yellow
γ = 62.496 (5)°	0.30 × 0.30 × 0.25 mm
*V* = 797.22 (12) Å^3^	

### Data collection

**Table:**

Bruker SMART 1000 CCD area-detector diffractometer	3368 independent reflections
Radiation source: normal-focus sealed tube	3152 reflections with *I* > 2σ(*I*)
graphite	*R*_int_ = 0.022
φ and ω scans	θ_max_ = 27.0°, θ_min_ = 2.0°
Absorption correction: multi-scan (*SADABS*; Sheldrick, 1996)	*h* = −11→11
*T*_min_ = 0.484, *T*_max_ = 0.535	*k* = −12→12
6168 measured reflections	*l* = −15→15

### Refinement

**Table:**

Refinement on *F*^2^	Secondary atom site location: difference Fourier map
Least-squares matrix: full	Hydrogen site location: mixed
*R*[*F*^2^ > 2σ(*F*^2^)] = 0.029	H-atom parameters constrained
*wR*(*F*^2^) = 0.077	*w* = 1/[σ^2^(*F*_o_^2^) + (0.040*P*)^2^ + 1.*P*] where *P* = (*F*_o_^2^ + 2*F*_c_^2^)/3
*S* = 1.05	(Δ/σ)_max_ < 0.001
3368 reflections	Δρ_max_ = 0.66 e Å^−^^3^
173 parameters	Δρ_min_ = −0.82 e Å^−^^3^
0 restraints	Extinction correction: *SHELXL*, Fc^*^=kFc[1+0.001xFc^2^λ^3^/sin(2θ)]^-1/4^
Primary atom site location: structure-invariant direct methods	Extinction coefficient: 0.0179 (16)

### Special details

**Table:**

Geometry. All e.s.d.'s (except the e.s.d. in the dihedral angle between two l.s. planes) are estimated using the full covariance matrix. The cell e.s.d.'s are taken into account individually in the estimation of e.s.d.'s in distances, angles and torsion angles; correlations between e.s.d.'s in cell parameters are only used when they are defined by crystal symmetry. An approximate (isotropic) treatment of cell e.s.d.'s is used for estimating e.s.d.'s involving l.s. planes.
Refinement. Refinement of *F*^2^ against ALL reflections. The weighted *R*-factor *wR* and goodness of fit *S* are based on *F*^2^, conventional *R*-factors *R* are based on *F*, with *F* set to zero for negative *F*^2^. The threshold expression of *F*^2^ > σ(*F*^2^) is used only for calculating *R*-factors(gt) *etc*. and is not relevant to the choice of reflections for refinement. *R*-factors based on *F*^2^ are statistically about twice as large as those based on *F*, and *R*- factors based on ALL data will be even larger.

### Fractional atomic coordinates and isotropic or equivalent isotropic displacement parameters (Å^2^)

**Table:**

	*x*	*y*	*z*	*U*_iso_*/*U*_eq_	
Zn1	0.77593 (3)	0.86751 (3)	0.75845 (2)	0.00894 (10)	
Cl1	0.97516 (7)	0.91131 (8)	0.83034 (6)	0.01767 (14)	
Cl2	0.74022 (9)	0.66200 (8)	0.92201 (5)	0.01983 (15)	
Cl3	0.86565 (9)	0.76054 (8)	0.61574 (5)	0.02216 (15)	
Cl4	0.51769 (7)	1.12209 (8)	0.66076 (6)	0.02434 (16)	
S1	−0.09151 (6)	0.72895 (7)	0.35203 (5)	0.00929 (13)	
S2	0.63717 (7)	0.43870 (7)	0.16847 (5)	0.01112 (13)	
N1	0.0383 (2)	0.6517 (3)	0.17917 (18)	0.0122 (4)	
H1A	0.0506	0.6334	0.1118	0.015*	
N2	0.4708 (3)	0.3426 (3)	0.33075 (19)	0.0131 (4)	
H2A	0.4395	0.2785	0.3941	0.016*	
N3	−0.2658 (3)	0.7787 (3)	0.1413 (2)	0.0169 (4)	
H3A	−0.3569	0.8179	0.1731	0.020*	
H3B	−0.2635	0.7475	0.0846	0.020*	
N4	0.7579 (3)	0.1184 (3)	0.3653 (2)	0.0194 (4)	
H4A	0.8469	0.0961	0.3298	0.023*	
H4B	0.7476	0.0411	0.4354	0.023*	
C1	−0.1143 (3)	0.7216 (3)	0.2097 (2)	0.0111 (4)	
C2	0.1792 (3)	0.6012 (3)	0.2685 (2)	0.0106 (4)	
C3	0.1324 (3)	0.6365 (3)	0.3667 (2)	0.0101 (4)	
C4	0.6262 (3)	0.2791 (3)	0.3020 (2)	0.0118 (4)	
C5	0.3552 (3)	0.5195 (3)	0.2475 (2)	0.0111 (4)	
C6	0.4236 (3)	0.5932 (3)	0.1553 (2)	0.0099 (4)	
C7	0.2430 (3)	0.6131 (3)	0.4750 (2)	0.0159 (5)	
H7A	0.3644	0.5591	0.4682	0.024*	
H7B	0.2304	0.5370	0.5564	0.024*	
H7C	0.2066	0.7276	0.4715	0.024*	
C8	0.3418 (3)	0.7794 (3)	0.0535 (2)	0.0145 (5)	
H8A	0.2226	0.8453	0.0610	0.022*	
H8B	0.3411	0.7821	−0.0309	0.022*	
H8C	0.4081	0.8325	0.0638	0.022*	

### Atomic displacement parameters (Å^2^)

**Table:**

	*U*^11^	*U*^22^	*U*^33^	*U*^12^	*U*^13^	*U*^23^
Zn1	0.01114 (14)	0.00947 (15)	0.00651 (15)	−0.00633 (11)	0.00174 (10)	−0.00251 (11)
Cl1	0.0133 (3)	0.0225 (3)	0.0240 (3)	−0.0108 (2)	0.0033 (2)	−0.0142 (3)
Cl2	0.0385 (3)	0.0201 (3)	0.0117 (3)	−0.0224 (3)	0.0131 (2)	−0.0079 (2)
Cl3	0.0459 (4)	0.0147 (3)	0.0096 (3)	−0.0169 (3)	0.0118 (3)	−0.0070 (2)
Cl4	0.0107 (3)	0.0210 (3)	0.0218 (3)	−0.0008 (2)	0.0008 (2)	−0.0011 (2)
S1	0.0094 (2)	0.0116 (3)	0.0101 (3)	−0.0057 (2)	0.00321 (19)	−0.0072 (2)
S2	0.0108 (2)	0.0074 (3)	0.0111 (3)	−0.0043 (2)	0.00468 (19)	−0.0010 (2)
N1	0.0165 (9)	0.0154 (10)	0.0116 (9)	−0.0103 (8)	0.0072 (7)	−0.0096 (8)
N2	0.0187 (9)	0.0095 (9)	0.0126 (9)	−0.0087 (8)	0.0098 (8)	−0.0047 (8)
N3	0.0162 (9)	0.0258 (11)	0.0145 (10)	−0.0111 (8)	0.0032 (8)	−0.0134 (9)
N4	0.0217 (10)	0.0070 (9)	0.0184 (10)	−0.0043 (8)	0.0096 (8)	−0.0001 (8)
C1	0.0161 (10)	0.0096 (10)	0.0106 (10)	−0.0081 (9)	0.0045 (8)	−0.0052 (8)
C2	0.0124 (10)	0.0098 (10)	0.0135 (11)	−0.0081 (8)	0.0058 (8)	−0.0059 (9)
C3	0.0107 (9)	0.0075 (10)	0.0143 (11)	−0.0058 (8)	0.0029 (8)	−0.0054 (8)
C4	0.0168 (10)	0.0091 (10)	0.0110 (10)	−0.0076 (9)	0.0059 (8)	−0.0047 (9)
C5	0.0128 (10)	0.0099 (10)	0.0150 (11)	−0.0072 (8)	0.0056 (8)	−0.0077 (9)
C6	0.0101 (9)	0.0085 (10)	0.0116 (10)	−0.0044 (8)	0.0018 (8)	−0.0053 (9)
C7	0.0121 (10)	0.0199 (12)	0.0187 (12)	−0.0077 (9)	0.0019 (9)	−0.0116 (10)
C8	0.0125 (10)	0.0106 (11)	0.0130 (11)	−0.0035 (9)	0.0001 (9)	−0.0016 (9)

### Geometric parameters (Å, °)

**Table:**

Zn1—Cl1	2.2531 (6)	N3—H3B	0.8322
Zn1—Cl3	2.2642 (6)	N4—C4	1.317 (3)
Zn1—Cl2	2.2707 (6)	N4—H4A	0.8693
Zn1—Cl4	2.2788 (6)	N4—H4B	0.8932
S1—C1	1.720 (2)	C2—C3	1.340 (3)
S1—C3	1.746 (2)	C2—C5	1.466 (3)
S2—C4	1.729 (2)	C3—C7	1.495 (3)
S2—C6	1.752 (2)	C5—C6	1.340 (3)
N1—C1	1.330 (3)	C6—C8	1.497 (3)
N1—C2	1.403 (3)	C7—H7A	0.9800
N1—H1A	0.8730	C7—H7B	0.9800
N2—C4	1.334 (3)	C7—H7C	0.9800
N2—C5	1.402 (3)	C8—H8A	0.9800
N2—H2A	0.8877	C8—H8B	0.9800
N3—C1	1.324 (3)	C8—H8C	0.9800
N3—H3A	0.8747		
			
Cl1—Zn1—Cl3	110.69 (3)	N1—C2—C5	119.60 (19)
Cl1—Zn1—Cl2	110.34 (2)	C2—C3—C7	128.8 (2)
Cl3—Zn1—Cl2	106.06 (2)	C2—C3—S1	110.42 (16)
Cl1—Zn1—Cl4	111.25 (3)	C7—C3—S1	120.71 (16)
Cl3—Zn1—Cl4	108.19 (3)	N4—C4—N2	126.2 (2)
Cl2—Zn1—Cl4	110.16 (3)	N4—C4—S2	122.76 (17)
C1—S1—C3	91.07 (10)	N2—C4—S2	110.99 (17)
C4—S2—C6	91.00 (11)	C6—C5—N2	113.51 (19)
C1—N1—C2	114.04 (18)	C6—C5—C2	127.9 (2)
C1—N1—H1A	123.1	N2—C5—C2	118.63 (19)
C2—N1—H1A	122.8	C5—C6—C8	128.4 (2)
C4—N2—C5	114.14 (18)	C5—C6—S2	110.35 (17)
C4—N2—H2A	123.8	C8—C6—S2	121.27 (16)
C5—N2—H2A	121.9	C3—C7—H7A	109.5
C1—N3—H3A	117.0	C3—C7—H7B	109.5
C1—N3—H3B	116.7	H7A—C7—H7B	109.5
H3A—N3—H3B	123.6	C3—C7—H7C	109.5
C4—N4—H4A	114.3	H7A—C7—H7C	109.5
C4—N4—H4B	119.7	H7B—C7—H7C	109.5
H4A—N4—H4B	125.8	C6—C8—H8A	109.5
N3—C1—N1	125.5 (2)	C6—C8—H8B	109.5
N3—C1—S1	123.29 (17)	H8A—C8—H8B	109.5
N1—C1—S1	111.22 (16)	C6—C8—H8C	109.5
C3—C2—N1	113.22 (18)	H8A—C8—H8C	109.5
C3—C2—C5	127.2 (2)	H8B—C8—H8C	109.5
			
C2—N1—C1—N3	−179.8 (2)	C6—S2—C4—N4	−178.7 (2)
C2—N1—C1—S1	0.0 (2)	C6—S2—C4—N2	0.43 (18)
C3—S1—C1—N3	−179.3 (2)	C4—N2—C5—C6	−1.1 (3)
C3—S1—C1—N1	0.85 (17)	C4—N2—C5—C2	178.5 (2)
C1—N1—C2—C3	−1.2 (3)	C3—C2—C5—C6	−112.4 (3)
C1—N1—C2—C5	179.0 (2)	N1—C2—C5—C6	67.4 (3)
N1—C2—C3—C7	−175.7 (2)	C3—C2—C5—N2	68.0 (3)
C5—C2—C3—C7	4.1 (4)	N1—C2—C5—N2	−112.2 (2)
N1—C2—C3—S1	1.8 (2)	N2—C5—C6—C8	−177.4 (2)
C5—C2—C3—S1	−178.40 (18)	C2—C5—C6—C8	3.0 (4)
C1—S1—C3—C2	−1.52 (18)	N2—C5—C6—S2	1.3 (2)
C1—S1—C3—C7	176.21 (19)	C2—C5—C6—S2	−178.20 (18)
C5—N2—C4—N4	179.3 (2)	C4—S2—C6—C5	−1.01 (17)
C5—N2—C4—S2	0.2 (2)	C4—S2—C6—C8	177.88 (18)

### Hydrogen-bond geometry (Å, °)

**Table:**

*D*—H···*A*	*D*—H	H···*A*	*D*···*A*	*D*—H···*A*
N1—H1A···Cl2^i^	0.87	2.78	3.440 (3)	133
N2—H2A···Cl4^ii^	0.89	2.79	3.487 (2)	137
N3—H3A···Cl4^iii^	0.87	2.50	3.322 (3)	156
N3—H3B···Cl2^iv^	0.83	2.36	3.196 (3)	179
N4—H4A···Cl1^v^	0.87	2.44	3.280 (3)	162
N4—H4B···Cl3^ii^	0.89	2.38	3.189 (2)	151

Symmetry codes: (i) −*x*+1, −*y*+1, −*z*+1; (ii) *x*, *y*−1, *z*; (iii) −*x*, −*y*+2, −*z*+1; (iv) *x*−1, *y*, *z*−1; (v) −*x*+2, −*y*+1, −*z*+1.
